# 

**Published:** 1895-09

**Authors:** 


### CONTENTS.

ORIGINAL ARTICLES—

Pyæmia and Septicæmia, in their Surgical Aspects. By J. McFadden Gaston, M. D., Atlanta, Ga ........................ 445

The Increase of Insanity and Some of its Causes. By S. E. Smith, M. S., M. D., Richmond, Ind ........................ 455

New York Letter ........................ 467

SELECTIONS AND ABSTRACTS—

The Care of the Navel ........................ 471

An Unjustifiable Operation ........................ 473

EDITORIAL—

Physicians and Life Insurance Companies........ 475
Notes........ 478
Book Reviews........ 483
Pamphlets Received........ 484
Special Notes........ 486

